# Cytochrome P450 (CYP450) Interactions Involving Atypical Antipsychotics Are Common in Community-Dwelling Older Adults Treated for Behavioral and Psychological Symptoms of Dementia

**DOI:** 10.3390/pharmacy8020063

**Published:** 2020-04-08

**Authors:** Adriana Matos, Kevin T. Bain, David L. Bankes, Anna Furman, Briana Skalski, James Verzicco, Jacques Turgeon

**Affiliations:** 1 Applied Precision Pharmacotherapy Institute, Tabula Rasa HealthCare, Moorestown, NJ 08057, USA; KBain0225@gmail.com (K.T.B.); DBankes@trhc.com (D.L.B.); AFurman@trhc.com (A.F.); 2 Learning and Development, Tabula Rasa HealthCare, Moorestown, NJ 08057, USA; BSkalski@trhc.com; 3 HealthCare Analytics, Tabula Rasa HealthCare, Moorestown, NJ 08057, USA; JVerzicco@trhc.com; 4 Precision Pharmacotherapy Research and Development Institute, Tabula Rasa HealthCare, Orlando, FL 32827, USA; JTurgeon@trhc.com

**Keywords:** antipsychotics, drug-drug interactions, older adults, PACE

## Abstract

Treatment of behavioral and psychological symptoms of dementia (BPSD) and comorbidities often necessitates the concomitant use of antipsychotics and non-antipsychotic drugs, thereby potentiating the risk for drug–drug interactions (DDIs). The primary objective of our study was to identify potentially clinically relevant cytochrome P450 (CYP)-mediated DDIs involving antipsychotics among participants enrolled in the Program of All-Inclusive Care for the Elderly (PACE) with BPSD. Additionally, we wanted to determine the prevalence of antipsychotic use in this population. The study included 10,001 PACE participants. The practice setting used a proprietary clinical decision support system (CDSS) to analyze simultaneous multidrug interactions. A retrospective analysis of pharmacy claims data was conducted to identify DDIs involving antipsychotics prescribed for BPSD, using snapshots of medication profiles paired with the CDSS. Of the participants who met inclusion criteria, 1190 (11.9%) were prescribed an antipsychotic; of those, 1071 (90.0%) were prescribed an atypical antipsychotic. Aripiprazole commonly caused (being a perpetrator drug 94.6% of the time) potential DDIs with antidepressants (e.g., duloxetine, venlafaxine, mirtazapine), opioids (e.g., hydrocodone, oxycodone, tramadol) and metoprolol via the CYP2D6 isoform. Risperidone commonly caused (85.7%) potential DDIs with donepezil, lamotrigine and trazodone via the CYP3A4 isoform. Quetiapine exclusively suffered (100%) from potential DDIs with amlodipine, buspirone, omeprazole or topiramate via the CYP3A4 isoform. Antipsychotics are commonly prescribed to PACE participants for BPSD treatment and they may interact with other drugs used to treat comorbidities. A thorough review of concomitant medications will help mitigate the likelihood of potentially dangerous CYP-mediated DDIs involving antipsychotics.

## 1. Introduction

Antipsychotic use among older Americans has been well studied, particularly in long-term care settings such as nursing homes [1,2]. Findings from a large population-based study, for instance, indicated that in 2012 approximately 33% of Medicare beneficiaries with dementia who resided in nursing homes were prescribed antipsychotics [2]. Based on these and similar findings, coupled with safety issues and warnings about using antipsychotics in older adults, agencies within the Department of Health and Human Services (HHS) have taken several actions to evaluate and reduce antipsychotic use among nursing home residents. However, similar actions for older adults receiving care in other settings, such as community-based practice, are lacking. 

Antipsychotics are approved by the U.S. Food and Drug Administration (FDA) and used for the treatment of psychiatric conditions (e.g., schizophrenia and bipolar disorder). However, in the older adult population, antipsychotics are commonly used off-label for treatment of behavioral and psychological symptoms of dementia (BPSD) [2,3,4]. Yet, there is a dearth of high-quality evidence demonstrating benefits of using antipsychotics for this indication [3,4,5]. Moreover, these medications are associated with substantial harms and risks including, for most of them, the risk of drug-induced Long QT Syndrome [3,4,5]. 

Treatment of BPSD and comorbidities often necessitates the concomitant use of antipsychotics and non-antipsychotic drugs, thereby potentiating the risk for drug–drug interactions (DDIs). Most antipsychotics are metabolized by the cytochrome P450 (CYP) enzymatic system. Interference with this system further exacerbates the risk for DDIs [6]. These interactions may cause or contribute significantly to poor outcomes such as treatment failures, hospital admissions, increased financial burdens to the health care system, and deaths [6,7,8]. Since antipsychotic-involved DDIs are frequently overlooked as causative or contributive factors, clinicians must be vigilant if prescribing antipsychotics to avoid unnecessary harm [6]. 

The primary objective of this study was to identify potentially clinically relevant DDIs involving antipsychotics among a nationally representative sample of nursing home eligible older adults in a community-based practice setting known as the Program of All-Inclusive Care for the Elderly (PACE). Secondary objectives were to determine the prevalence of antipsychotic use and describe other drugs commonly co-prescribed with antipsychotics. To achieve these objectives, pharmacy claims data were used for PACE participants who were prescribed an antipsychotic for treatment of BPSD.

## 2. Materials and Methods 

### 2.1. Study Protocol and Practice Description

The Biomedical Research Alliance of New York (BRANY), an independent Institutional Review Board (IRB), reviewed the study protocol and gave exempt status (approval number 18-12-228-427). Given the retrospective nature of collecting existing data, the IRB waived informed consent. Notwithstanding, the researchers provided evidence of training in human subject research and conducted this study in compliance with the HIPAA Privacy Rule. The study was registered with ClinicalTrials.gov (NCT03692182). 

The practice setting was a centralized pharmacy that services >25% of PACE participants in the United States. The dispensing of all medications for PACE participants, including over-the-counter products and medications obtained by on-site fulfillment (e.g., automated dispensing machines) and by local procurement (i.e., community pharmacy networks), is managed by this centralized pharmacy and documented in its electronic record. Additionally, this pharmacy provides medication therapy management services, including comprehensive and targeted medication safety reviews, which consider simultaneous multidrug interactions and time-of-day administrations to optimize drug regimens for PACE participants [9]. The pharmacy is also a training site for pharmacy students and postgraduate pharmacy residents.

### 2.2. Study Population

PACE is a federally funded program that provides comprehensive medical and supportive services to participants 55 years and older who are certified by their states as needing nursing home care but are able to live in the community, as an alternative to institutionalization. The average PACE participant is 76 years old with 6 chronic health conditions (e.g., dementia, diabetes), and the majority (70%) are female. Services available to PACE participants include, but are not limited to, pharmacy, primary care, physical therapy, and others that are determined to be necessary by an interdisciplinary healthcare team. Medicare and Medicaid cover these services via capitated financing, allowing PACE organizations to cover any service deemed necessary for optimal participant care. PACE organizations assume full financial risk for participant outcomes. Additional information regarding PACE has been described extensively elsewhere [9,10,11,12,13].

The study population included participants enrolled in PACE who received their medications from the pharmacy and its network pharmacy services. Using claims data for the calendar year 2017, monthly snapshots of medication regimens were taken to determine patterns of medication use as a function of time. Medication use did not significantly fluctuate from month to month. Therefore, a snapshot of a single month as the index point was used in the analyses.

### 2.3. Analytical Sample

The analytical sample of interest was PACE participants using an antipsychotic during the index month. Because data were collected retrospectively, prescription of an antipsychotic served as a proxy for use. Participants with a diagnosis of bipolar disorder or schizophrenia, according to documented medication indications in the pharmacy records, were excluded from the sample. The final analytical sample included PACE participants who were prescribed an antipsychotic for all other documented diagnoses that could be associated with BPSD (e.g., agitation, anxiety, psychosis), which were documented in the pharmacy records. Bipolar disorder or schizophrenia are known FDA-approved indications for antipsychotics, whereas other documented indications are or possibly could be manifestations of BPSD [2,3].

### 2.4. Definining Potentially Clinically Signifcant DDIs

The researchers aimed at identifying potentially clinically relevant DDIs involving antipsychotics and concomitantly used drugs. However, based on the retrospective nature of this study, the researchers were unable to assess drug concentrations, therapeutic drug monitoring or patient reported consequences of the potential DDIs. In lieu of these shortcomings, the researchers aimed to assess potentially clinically relevant DDIs utilizing data available within a proprietary clinical decision support system (CDSS) [14,15,16]. The software includes the relative contribution CYP isoforms and metabolic pathways as well as relative isoform affinities for all drugs to assess competitive inhibition between substrates of a CYP isoform, non-competitive inhibition between substrates and inhibitors, and induction between substrates and inducers. From these data, the drug(s) involved in potentially clinically relevant DDIs which were “causing” or “suffering from” the interactions were determined. Therefore, the researchers utilized a drug’s metabolic pathway with a threshold of 30% as a proxy to determine clinical significance, as inhibition of such a disposition process shall be expected to lead to significant increase in area under the curve (AUC) or the maximum plasma concentration (Cmax). This threshold was based on a conservative FDA bioequivalence standard of 30%, as well as published pharmacokinetic data [16,17].

### 2.5. Assessing Potentially Clinically Signifcant DDIs

For the analytical sample, co-prescribed drugs were determined by extracting participants’ drug regimens during the index month. These data were reported as numbers and percentages. The active ingredient for each drug, including combination products, was extracted based on a unique National Drug Code (NDC) [18]. Drug dosage formulations for non-oral routes of administration (e.g., intramuscular) were included in the prevalence analysis but excluded from the potential DDI analysis since these drugs do not undergo first-pass metabolism through the CYP enzymatic system [19]. Time of administration (TOA) was denoted in binary code, with “1” denoting administered at the same time of day and “0” denoting separate TOA. The sum of TOA was calculated for the specific antipsychotic with each competing drug; for multiple competing drugs, this analysis was repeated. If the sum totaled “2” at any time of day, then the determined potential DDI carried forward in the analysis as a Level 2 interaction.

Each active ingredient was then assigned the CYP isoform(s) corresponding to its metabolic pathway [20]. For each CYP isoform-active ingredient pair, a ranking *a priori* was determined, such that an inhibitor, inducer, strong affinity substrate, moderate affinity substrate and weak affinity substrate received a score of “5” through “1”, respectively. DDIs between two similar affinity substrates for each CYP isoform were excluded due to the reduced likelihood of resulting in a potentially clinically significant interaction. Finally, the percent metabolic pathway was assigned to each active ingredient that was a substrate of a CYP isoform; in the case of multiple metabolic pathways per active ingredient, the percent contribution of each CYP isoform was likewise assigned.

When a potential DDI was determined (i.e., Level 2), the predetermined affinities for a specific CYP isoform were used to determine whether the drug “caused” or “suffered from” the interaction. Respectively, the drugs were denoted as either “perpetrator” or “victim” of the interaction. If the result of Level 2 was a “tie”, whereby both drugs had a similar affinity towards a particular CYP isoenzyme, then the DDI was excluded from the analysis. The rules used to identify whether the antipsychotic was either the perpetrator or victim of the interaction can be found in Table A1 located in Appendix A.

The final DDI analysis (i.e., Level 3) was based on results of the Level 2 interactions. Specifically, if Level 2 resulted in a “win”, and the metabolic pathway of the “victim” was ≥30%, then the antipsychotic was the “perpetrator”. Conversely, if the metabolic pathway of the “victim” was <30%, then the interaction was “not concerned”. If Level 2 resulted in a “loss”, and the metabolic pathway was ≥30%, then the antipsychotic was the “victim”. If Level 2 resulted in a “loss”, and the metabolic pathway was <30%, then the interaction was “not concerned”. 

## 3. Results

**Figure 1:**
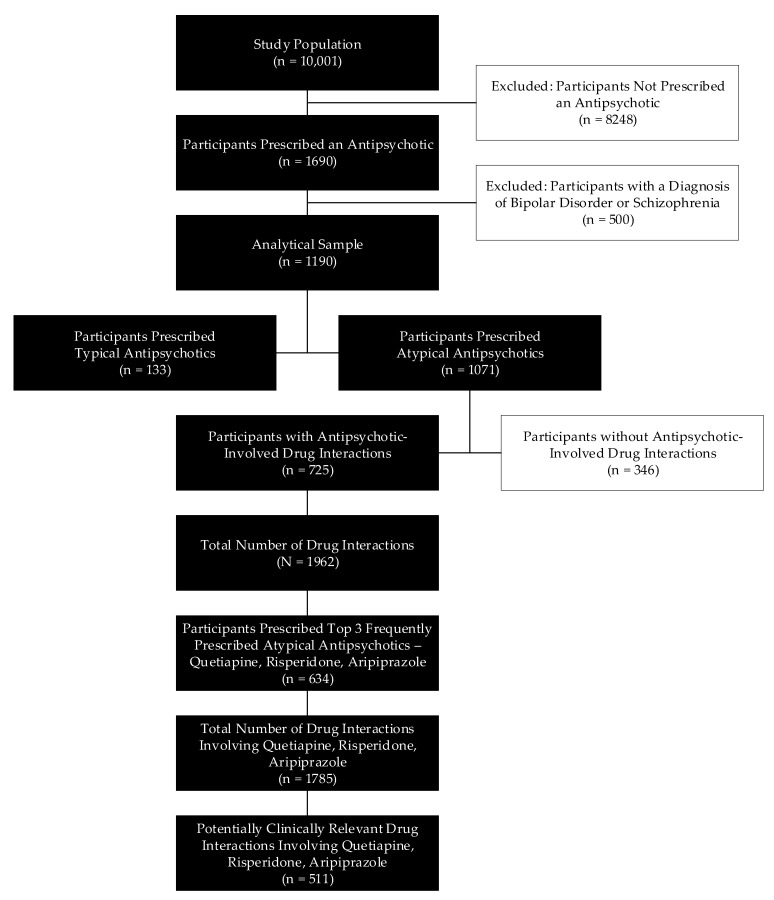
Participant identification flowchart. The study population was comprised of all Program of All-Inclusive Care for the Elderly (PACE) participants who received their medications from the pharmacy. The analytical sample consisted of PACE participants who were prescribed an antipsychotic for a documented indication of behavioral and psychological symptoms of dementia (BPSD). Fourteen participants were prescribed both a typical and atypical antipsychotic.

Figure 1 shows the process of sampling the study population. During the index month, 10,001 participants were enrolled in PACE programs that received pharmacy services from CareKinesis. Among the study population, 1690 (16.9%) were prescribed an antipsychotic for any indication. After exclusion criteria were applied, the analytical sample included 1190 participants, or 11.9% of the study population, who were prescribed at least one antipsychotic for treatment of BPSD. A relatively small number of participants (n = 14) were concurrently prescribed both a typical (first generation) and atypical (second generation) antipsychotic. Of those included in the analytical sample, the overwhelming majority (90.0%) were prescribed an atypical antipsychotic. Because of such high prevalence of atypical antipsychotic use, the rest of the analyses focused on this class of antipsychotics. 

In total, the CDSS detected 1962 DDIs, occurring among 725 PACE participants, or 67.7% of the analytical sample prescribed atypical antipsychotics. The top three most frequently prescribed atypical antipsychotics were quetiapine (n = 530), risperidone (n = 257) and aripiprazole (n = 157), representing 44.5%, 21.6% and 13.2% of the analytical sample, respectively. Among the patients taking quetiapine, risperidone, or aripiprazole, 1785 DDIs were identified, of which 511 (28.6%) were deemed to be potentially clinically relevant.

Table 1 presents the characteristics of the study population and analytical sample. The mean (± standard deviation) age of the study population was 76.6 (± 10.4) years, and the majority (67.7%) were female. Participants who were prescribed atypical antipsychotics were younger than those who were not prescribed antipsychotics (75.5 vs 77.1 years, *p* < 0.01); however, the differences in age were not clinically significant. The gender distribution was relatively similar across groups; however, there was a trend towards a larger proportion of atypical antipsychotic users being female (*p* = 0.10).

Table 2 reports the frequency of potentially clinically relevant DDIs associated with the respective atypical antipsychotic and its associated metabolic pathway. Among these atypical antipsychotics, potentially clinically relevant DDIs were most prevalent among participants prescribed risperidone, occurring in 46.1% of prescriptions. Risperidone more frequently caused potentially clinically relevant DDIs (85.7%) than suffered from them (14.3%). Through the CYP3A4 metabolic pathway, risperidone (a moderate affinity substrate) usually caused (89.2%) potentially clinically relevant DDIs and only suffered from 10.8% of these DDIs. Through the CYP2D6 metabolic pathway, risperidone (also a moderate affinity substrate) usually caused (78.8%) potentially clinically relevant DDIs and only suffered from 21.2% of these DDIs. Aripiprazole (a high affinity substrate) commonly caused CYP2D6-mediated drug interactions (94.6%). Quetiapine (a weak affinity substrate) exclusively suffered from potential DDIs via the CYP3A4 isoform (100%).

Examples of co-prescribed medications involved in potentially clinically relevant DDIs with the top three prescribed atypical antipsychotics are displayed in Table 3. Through the CYP3A4 metabolic pathway, risperidone most commonly caused potential DDIs with donepezil, lamotrigine and trazodone, whereas risperidone commonly suffered from potential DDIs with topiramate, phenobarbital and diltiazem. Through the CYP2D6 metabolic pathway, risperidone commonly caused potential DDIs with donepezil, antidepressants (e.g., mirtazapine, venlafaxine) as well as opioids (e.g., tramadol, oxycodone, hydrocodone), whereas risperidone most commonly suffered from potential DDIs with antidepressants (e.g., bupropion, paroxetine, fluoxetine). Aripiprazole commonly caused CYP2D6-mediated DDIs with antidepressants (e.g., duloxetine, venlafaxine, mirtazapine), opioids (e.g., hydrocodone, oxycodone, tramadol) and metoprolol. Quetiapine frequently suffered from potential DDIs via the CYP3A4 isoform with amlodipine, buspirone, omeprazole, and topiramate.

## 4. Discussion

This research found that approximately 12% of PACE participants were prescribed antipsychotics for BPSD, which is slightly below the national average for community-dwelling older adults (i.e., about 14%), yet below the average for nursing home residents (i.e., about 16% in 2017) [2,21]. About two-thirds of PACE participants who took an atypical antipsychotic for BPSD had a drug interaction and three atypicals (quetiapine, risperidone, and aripiprazole) were taken by nearly 90% of these participants. These three atypical antipsychotics are specifically mentioned by clinical practice guidelines and are commonly used among patients with BPSD [22]. Moreover, these three drugs accounted for the disproportionate amount, about 90%, of interactions identified among atypical antipsychotic users and of their interactions, nearly 30% were deemed clinically significant. Collectively, these results suggest that drug interactions should be a consideration when selecting an atypical antipsychotic therapy for BPSD.

### 4.1. Quetiapine: Interactions and Implications

Quetiapine exclusively suffered from potential DDIs through the CYP3A4 metabolic pathway. This is unsurprising. It is well established that quetiapine is a weak substrate (low affinity) of CYP3A4 and, in general, most PK interactions involve this isoform [16,20]. The risk for QT interval prolongation is the major potential consequence with these DDIs. It is well known that quetiapine prolongs the QT interval in a dose-dependent manner [23]. Because DDIs that increase quetiapine’s plasma concentration, such as those occurring with other CYP3A substrates like amlodipine and sertraline [23,24], can mimic higher doses of quetiapine, it is likely that they cause or contribute to the associated risk for QT interval prolongation. Two case reports from the literature of CYP3A4-mediated DDIs involving quetiapine resulted in TdP. One report involved concurrent use of the CYP3A4 inhibitor fluvoxamine, while the other involved concomitant use of the CYP3A4 competitive higher affinity substrate lovastatin [25,26].

While monitoring the QT interval is certainly prudent when such interactions exist, in actuality, the prevalence of interactions should call quetiapine’s role in BPSD management into further question. Current clinical practice guidelines do not recommend quetiapine given a lack of demonstrated efficacy to balance against the well-established, life threatening risks [4,5]. While quetiapine may be preferred in patients with movement disorders, the emergence of pimavancerin could supplant quetiapine’s niche role for this subset of patients in the future [27]. Preliminary studies suggest pimavanserin may significantly reduce symptoms of psychosis over the short term (i.e., 6–12 weeks) compared to placebo [28,29]. Like quetiapine, pimavancerin is a CYP3A4 substrate and can prolong the QT interval. While similar interactions and subsequent consequences might be expected, [30] efficacy benefits may justify risks of these interactions. Future head-to-head studies with quetiapine will be needed to fully assess these agents’ place in therapy [31].

### 4.2. Risperidone: Interactions and Implications

In the analytic sample of PACE participants, risperidone frequently caused potential DDIs, most commonly through the CYP3A4 and CYP2D6 metabolic pathways and repeatedly with donepezil and several antidepressants (e.g., trazodone, mirtazapine, venlafaxine). Both risperidone and donepezil are metabolized by the CYP isoforms 3A4 and 2D6 [32,33,34]. Risperidone is a moderate affinity substrate, whereas donepezil is a weak affinity substrate of these isoforms [34,35,36,37]. When taken concomitantly, risperidone is expected to competitively inhibit donepezil’s metabolism, thereby increasing its plasma concentration. Drug interaction studies demonstrated increases in donepezil’s plasma concentration during co-administration with inhibitors of the 3A4 isoform, such as cimetidine [34] and ketoconazole [36].

This raises the vexing question: might certain risperidone-involved DDIs benefit some patients suffering from BPSD? Specifically, are the benefits of risperidone related to its antipsychotic effects or to the boosted effects of donepezil resulting from this DDI—or perhaps both? Does the synergistic interaction increase the acetylcholinesterase inhibitor effect, thus allowing a lower effective dose of donepezil? Risperidone is the most well-studied antipsychotic for treatment of BPSD and is recommended as the drug of choice for BPSD [3,38,39]. Donepezil is the most commonly prescribed acetylcholinesterase inhibitor for treatment of dementia [39]. Although conflicting evidence exists, some studies have shown that monotherapy with donepezil is beneficial for treating certain behavioral symptoms of dementia, in addition to its primary effects on cognition and function [40,41]. In general, though, combination therapy with an acetylcholinesterase inhibitor and an antipsychotic tends to show better results than monotherapy for BPSD [41,42].

It is important to remember that DDIs mediated by CYP isoforms can mimic genetic polymorphisms [43,44]. Patients expressing phenotypic intermediate or poor metabolizer status for the *CYP2D6* gene responded better (e.g., had improved cognition scores) to donepezil than those expressing phenotypic normal metabolizer status [45,46]. Those expressing phenotypic ultra-rapid metabolizer status for the *CYP2D6* gene showed no clinical improvement with donepezil treatment [43,44,47]. Given the high likelihood that these two drugs are concomitantly prescribed for dementia treatment, this potential DDI warrants additional study, particularly with outcomes-based research.

Although risperidone did not frequently suffer from potential DDIs, when it did occur, paroxetine and fluoxetine were commonly associated with causing the interaction through the CYP2D6 metabolic pathway. Among the atypical antipsychotics, risperidone is associated with a possible dose-dependent effect for extrapyramidal symptoms (EPS), a high risk for causing hyperprolactinemia, and a moderate risk for causing weight gain, dyslipidemia, and hyperglycemia [5,48,49]. These risks may be further increased by reduced isoform activity, such as that resulting from CYP-mediated DDIs. For example, pharmacokinetic drug interaction studies demonstrated a significant increase in risperidone’s plasma concentration during co-administration with fluoxetine [49,50,51]. Patients developed Parkinsonian symptoms during the second week of co-administration [49]. Mechanistically, both risperidone and fluoxetine are metabolized by CYP2D6 [32,33,51,52], whereby risperidone is a moderate affinity substrate, while fluoxetine is a strong affinity substrate [35,53]. When taken concomitantly, fluoxetine is expected to competitively inhibit risperidone’s metabolism, thereby increasing its plasma concentration and potentially leading to EPS symptoms. Given that paroxetine is a high affinity substrate and a mechanism-based inhibitor of CYP2D6, similar results can be expected. Therefore, prescribing clinicians would be wise to consider alterative SSRIs that are not appreciably metabolized by or inhibit CYP2D6, such as sertraline.

### 4.3. Aripiprazole: Interactions and Implications

In the analytical sample, aripiprazole frequently caused potential DDIs, most commonly via the CYP2D6 metabolic pathway and with antidepressants such as mirtazapine, venlafaxine and duloxetine. Both aripiprazole and mirtazapine are metabolized by CYP2D6, whereby aripiprazole is a strong affinity substrate and mirtazapine is a weak affinity substrate [54]. When taken concomitantly, aripiprazole is expected to competitively inhibit mirtazapine’s metabolism, thereby increasing its plasma concentration. There are limited studies analyzing the pharmacokinetic interaction between these two drugs; however, a therapeutic drug monitoring service evaluated the effect of aripiprazole’s concentrations when co-administered with mirtazapine. As expected, mirtazapine did not significantly affect serum concentrations of aripiprazole or its metabolite, dehydroaripiprazole [55]. Unfortunately, the researchers did not analyze the serum concentration of mirtazapine.

Although aripiprazole may be considered due its potential effectiveness in BPSD as well as its relative milder adverse reaction profile compared to other atypical antipsychotics, it is prudent to evaluate how its addition to a patient’s drug regimen may exacerbate the risks associated with the victim drug [5,56,57]. With regards to mirtazapine, it is well known that the properties of the drug allow it to be considered in patients with depressive disorder and challenges with insomnia and/or weight loss. However, older adults appear to experience an inverse relationship between the dose of mirtazapine and sedation. Mirtazapine binds more to the histamine receptors at lower doses, which increases the risk for sedation. Conversely, mirtazapine binds more to the norepinephrine receptors at higher doses, which may interfere with sleep [58,59]. Therefore, while mirtazapine may be prescribed appropriately at lower doses to resolve insomnia, due to the presence of DDIs, the patient may unknowingly experience higher concentrations and potentially experience worsening insomnia. Overall, while these findings do not necessarily rule out a potential DDI between aripiprazole and mirtazapine, as well as other competing substrates, from a theoretical standpoint, the clinical relevance of such interactions merits further consideration by researchers and, perhaps, monitoring by clinicians.

### 4.4. Limitations

One limitation of this study is its retrospective nature. First, there is a risk of selection bias. Participants were identified using the diagnosis located in the prescription description from computerized pharmacy records. Moreover, participants may have been included while on antipsychotics for FDA-approved indications (e.g., major depressive disorder). Second, drug regimens were taken as a snapshot and thus interactions with temporal significance (e.g., *pro re nata* prescriptions) were not delineated out of the entire sample. Third, identifying drug interactions from computerized pharmacy records provides only an indirect measure of actual participant experiences. The data sources used did not capture actual plasma concentrations or allow determination of whether participants experienced effects—negative or positive—from identified potential DDIs. This study was not designed to detect the clinical consequences of DDIs or drug changes. Evidence-based information related to DDIs are oftentimes limited to case reports in healthy subjects, which results in challenges to support DDIs as described in this study. Ultimately, clinicians may be uncertain as to how to intervene or whether intervention is necessary given the lack of high-quality evidence. Moreover, evidence supports substantial variability in DDI decision support systems in determining DDIs that are clinically significant. Therefore, if this study was repeated utilizing other DDI software, results may differ. Additionally, this study was not designed to detect genetic variation, which may influence drug response. Fourth, participants in this study with an antipsychotic-involved DDI may have had their drug dose adjusted by our pharmacists in an effort to avoid an adverse interaction; however, this study did not assess such clinical outcomes. Fifth, these results might not be generalizable towards participants who are prescribed antipsychotics for indications other than BPSD, or those who may be residing in nursing home care. Lastly, DDIs directly involving active metabolite(s) were not included in the analysis.

## 5. Conclusions

Antipsychotics are commonly prescribed to PACE participants and may interact with other prescribed drugs used to treat comorbidities. Utilization of this medication class, especially in the older adult population, requires a thorough review of concomitant medications in order to mitigate the likelihood of potentially dangerous CYP-mediated DDIs.

## Figures and Tables

**Table 1:** Characteristics of the study population and analytical sample ^1^.

Characteristic	Study Population (N = 10,001)		Analytical Sample (N = 1190) ^2^		*p*-Value ^3^
	All Drug Users (n = 10,001)	Non-Antipsychotic Users (n = 8248)	Typical Antipsychotic Users (n = 133)	Atypical Antipsychotic Users (n = 1071)	
**Age (years)**	76.6 ± 10.4	77.1 ± 10.3	75.8 ± 10.5	75.5 ± 10.4	<0.01
**Gender**					0.10
**Male**	3240 (32.4)	2702 (32.8)	49 (36.8)	314 (29.3)	
**Female**	6771 (67.7)	5546 (67.2)	84 (63.2)	757 (70.7)	

^1^ Data are indicated as mean ± standard deviation or number (percentage). ^2^ A relatively small number of participants (n = 14) were prescribed both a typical and atypical antipsychotic. ^3^ Comparison between atypical antipsychotic users comprising the analytical sample and non-antipsychotic users.

**Table 2:** Most frequently prescribed atypical antipsychotics considering commonly co-prescribed medications ^1^.

Antipsychotic Involved DDIs ^2,3^	Top 3 Prescribed Atypical Antipsychotics		
	Quetiapine	Risperidone	Aripiprazole
Total Number of Patients with an AP Rx	530	257	157
Total Number of Patients with AP Rx with at least 1 DDI ^4^	334 (63.0)	174 (67.7)	130 (82.8)
Total Number of DDIs Identified ^5^	694	547	544
Total Number of Potentially Clinically Relevant DDIs ^6^	112 (16.1)	252 (46.1)	147 (27.0)
**CYP2D6**			
AP as Victim		18 (21.2)	8 (5.4)
AP as Perpetrator		67 (78.8)	139 (94.6)
**CYP3A4**			
AP as Victim	112 (100.0)	18 (10.8)	
AP as Perpetrator		149 (89.2)	

Abbreviations: AP = antipsychotic, CYP = cytochrome P450, DDI = drug–drug interaction, Rx = prescription. ^1^ Data are indicated as number (percentage). ^2^ Victim refers to the antipsychotic “suffering from” the potential DDI, whereas perpetrator refers to the antipsychotic “causing” the potential DDI. ^3^ Data are indicated as number of potential DDIs involving the antipsychotic through the specific metabolic pathway (percentage of CYP isoenzyme-mediated DDIs). For example, risperidone was involved in 85 potential DDIs mediated by the CYP2D6 isoenzyme, and was the victim in 18 (21.2%) of the interactions occurring via this metabolic pathway. ^4^ When summed, the total exceeds 634 patients. Three patients were concomitantly prescribed quetiapine and aripiprazole and one patient was concomitantly prescribed quetiapine and risperidone. ^5^ Total number of DDIs identified prior to Level 2 and Level 3 analysis. ^6^ Data are indicated as total number of potential DDIs involving the antipsychotic through all metabolic pathways (percentage of total antipsychotic prescriptions) post Level 3 analysis. For example, aripiprazole was involved in a total of 147 potentially clinically relevant DDIs, which entailed 27.0% of all interactions (n = 544) for aripiprazole; these potentially clinically relevant DDIs were mediated through the CYP2D6 isoenzyme, whereby aripiprazole was predominantly (94.6%) the perpetrator of the interaction.

**Table 3:** Potentially clinically relevant drug interactions between top 3 prescribed atypical antipsychotics and co-prescribed Medications.

**Atypical Antipsychotic**	**Co-Prescribed Medications ^1^**	**CYP450 Isoenzyme**
**Perpetrator ^2^**	**Victim ^3^**	
Aripiprazole	Duloxetine Hydrocodone Metoprolol Mirtazapine Oxycodone Tramadol Venlafaxine	2D6
Risperidone	Donepezil Hydrocodone Mirtazapine Oxycodone Tramadol Venlafaxine	2D6
	Alprazolam Clonazepam Donepezil Lamotrigine Trazodone Zolpidem	3A4
**Co-prescribed Medications**	**Atypical Antipsychotic**	**CYP450 Isoenzyme**
**Perpetrator**	**Victim**	
Amiodarone Paroxetine	Aripiprazole	2D6
Bupropion Fluoxetine Paroxetine	Risperidone	2D6
Carbamazepine Diltiazem Phenobarbital Primidone Topiramate		3A4
Amlodipine Buspirone Omeprazole Topiramate	Quetiapine	3A4

Abbreviations: CYP = cytochrome P450, DDI = drug–drug interaction. ^1^ Examples of co-prescribed medications involved in potentially clinically relevant DDIs associated with the top 3 prescribed atypical antipsychotics. For example, aripiprazole was the perpetrator of potential DDIs mediated by the CYP2D6 isoenzyme, and metoprolol was the victim, whereas paroxetine was the perpetrator of potential DDIs mediated by the CYP2D6 isoenzyme, and aripiprazole was the victim. ^2^ Perpetrator refers to the drug “causing” the potential DDI. ^3^ Victim refers to the drug “suffering from” the potential DDI.

## Appendix A

**Table A1:** Identification of Antipsychotics as either victim or perpetrator drugs.

Level	Property	Value	Outcome
**Level 2**	Affinity	Greater	Perpetrator ^a^
		Weaker	Victim ^b^
		Similar	Not Concerned ^c^
**Level 3**	Metabolic Pathway ≥30%	Yes	Confirm Outcome of Level 2 ^d^
		No	Not Concerned ^e^

Abbreviations: CYP = cytochrome, DDI = drug–drug interaction. ^a^ If the antipsychotic demonstrated greater affinity for the CYP isoenzyme compared to the competing drug, then the antipsychotic was the perpetrator, and the result was a “win”. ^b^ If the antipsychotic demonstrated weaker affinity for the CYP isoenzyme compared to the competing drug, then the antipsychotic was the victim, and the result was a “loss”. ^c^ If both the antipsychotic and the competing drug demonstrated similar affinity for the CYP isoenzyme, then the result was “not concerned”. ^d^ If the antipsychotic had ≥30% metabolic pathway, then the antipsychotic was either the perpetrator or victim of the potential DDI based on Level 2. ^e^ If the antipsychotic had <30% metabolic pathway, then the potential DDI was “not concerned”.
